# Supplementary material for: Clinically relevant atovaquone-resistant human malaria parasites fail to transmit by mosquito
Source: Nat Commun. 2023 Oct 12;14:6415. doi: 10.1038/s41467-023-42030-x (PMC10570281; doi:10.1038/s41467-023-42030-x)
Supplement: Supplementary file 1 — Supplementary Information [file 41467_2023_42030_MOESM1_ESM.pdf]

Supplementary Information for

Clinically relevant atovaquone-resistant human malaria parasites fail to transmit by mosquito

Victoria A. Balta[1,2], Deborah Stiffler[1,2], Abeer Sayeed[1,2], Abhai K. Tripathi[1,2], Rubayet Elahi[1,2], Godfree Mlambo[1,2], Rahul P. Bakshi[2,3], Amanda G. Dziedzic[1], Anne E. Jedlicka[1], Elizabeth Nenortas[3], Keyla Romero-Rodriguez[3], Matthew A. Canonizado[3], Alexis Mann[1,2], Andrew Owen[4], David J. Sullivan[1,2], Sean T. Prigge[1,2], Photini Sinnis[1,2], Theresa A. Shapiro[1,2,3,*]

[1]W. Harry Feinstone Department of Molecular Microbiology and Immunology, The Johns Hopkins Bloomberg School of Public Health, Baltimore, Maryland, 21205
[2]The Johns Hopkins Malaria Research Institute, Baltimore, MD, 21205
[3]Division of Clinical Pharmacology, Departments of Medicine and of Pharmacology and Molecular Sciences, The Johns Hopkins University, Baltimore, MD, 21205-2186
[4]Centre of Excellence in Long-acting Therapeutics (CELT), Department of Pharmacology and Therapeutics, University of Liverpool, Liverpool L69 3BX, UK

[*]Corresponding author, tshapiro@jhmi.edu

Supplementary Figures S1 to S11
Supplementary References

A

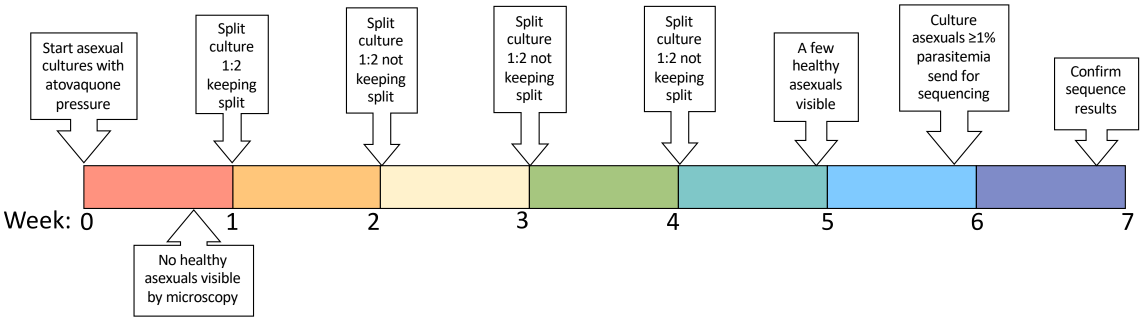

B

	Parent Strain	Starting Culture Volume (ml)	Hematocrit	Total # of RBCs	Starting Parasitemia	Total # parasites pressured	Atovaquone concentration (nM)	Successful isolates	Mutant cells first evident in culture
**Trial 1**	NF54	100	4%	$2.20x10^{10}$	3.0%	$6.60x10^{8}$	70	Y268S	day 34
**Trial 2**	NF54	100	4%	$2.20x10^{10}$	3.0%	$6.60x10^{8}$	35	L144S	day 29
**Trial 3**	NF54	100	4%	$2.20x10^{10}$	3.0%	$6.60x10^{8}$	20	Y268S	day 25
**Trial 4**	NF54	50	4%	$1.10x10^{10}$	3.0%	$3.30x10^{8}$	70	None	
**Trial 5**	NF54	100	2%	$1.10x10^{10}$	4.0%	$4.40x10^{8}$	70	None	
**Trial 6**	NF54	100	2%	$1.10x10^{10}$	4.5%	$4.95x10^{8}$	70	None	
**Trial 7**	NF54	50	4%	$1.10x10^{10}$	3.0%	$3.30x10^{8}$	35	None	
**Trial 8**	NF54	100	2%	$1.10x10^{10}$	3.0%	$3.30x10^{8}$	10	L144S	day 47
**Trial 9**	NF54	100	2%	$1.10x10^{10}$	5.0%	$5.50x10^{8}$	50	None	
**Trial 10**	NF54 C21	50	2%	$5.50x10^{9}$	7.4%	$4.07x10^{8}$	70	None	
**Trial 11**	NF54 C21	50	2%	$5.50x10^{9}$	6.0%	$3.30x10^{8}$	35	Y268S	day 35
**Trial 12**	NF54 C3	100	4%	$2.20x10^{9}$	6.0%	$1.32x10^{9}$	70	none	
**Trial 13**	NF54 C19	100	4%	$2.20x10^{9}$	6.0%	$1.32x10^{9}$	70	none	

C

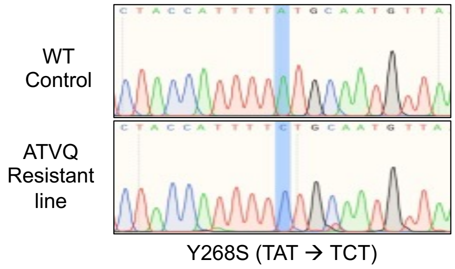

Supplementary Figure S1. ***In vitro*** **selection of gametocyte-competent, atovaquone-resistant,** ***P. falciparum*****.** **A** Representative timeline for selecting atovaquone-resistant parasites from under constant drug pressure. **B** Compilation of thirteen attempted selections. NF54 parent line was known to generate infectious gametocytes; parasites from trials 10-13 were low passage isolates from clinical trial volunteers[1]. Total number of parasites pressured was 7.8×10^9. **C** Sequence data revealing an A-to-C mutation resulting in Y268S change. No evidence for heteroplasmy was detected in any of the WT or mutant samples.

A

Primer Code	Sequence (5’ to 3’)[a]	Product (bp)	Primer Description
**Full length sequence of *Pfcytb* from cells that survive atovaquone pressure**			
CytBSeqF	GGTAATGCTGCCATTGATGTAGCATTAC	1848	Forward for amplification
CytBSeqR	GCATGCAATACCGAACATTTATCG		Reverse for amplification
CytBSeqF1	CGTTGGTTATGTCTTACCATGGGG	-	For sequencing
CytBSeqF2	GGAATTATACCTTTATCACATCCTGATAATGC	-	For sequencing
**Nested PCR of *Pfcytb* to detect parasite DNA in tissues**			
CytB1	CTCTATTAATTTAGTTAAAGCACA	939	Forward for primary PCR
CytB2	ACAGAATAATCTCTAGCACC		Reverse for primary PCR
CytB1.1	CGTTGGTTATGTCTTACCATGGGG	538	Forward for nested PCR
CytB2.2	AGTTGTTAAACTTCTTTGTTCTGC		Reverse for nested PCR
**RFLP to detect WT and mutant parasites in mixed infections**			
CytB1	CTCTATTAATTTAGTTAAAGCACA	939	Forward for primary PCR
CytB2	ACAGAATAATCTCTAGCACC		Reverse for primary PCR
CytB5	GGTTTACTTGGAACAGTTTTTAACAaTG	250	Reverse for secondary PCR of WT
CytB8	GTAGCACAAATCCTTTAGGGTATGA		Forward for both secondary PCRs
CytB9	GGTTTACTTGGAACAGTTTTTAACAcTG		Reverse for secondary PCR of mutant

[a]Residues in lower case red encode base changes to generate *Nsi*I (a) or *Pst*I (c) endonuclease sites that distinguish mutant from WT at codon 268.

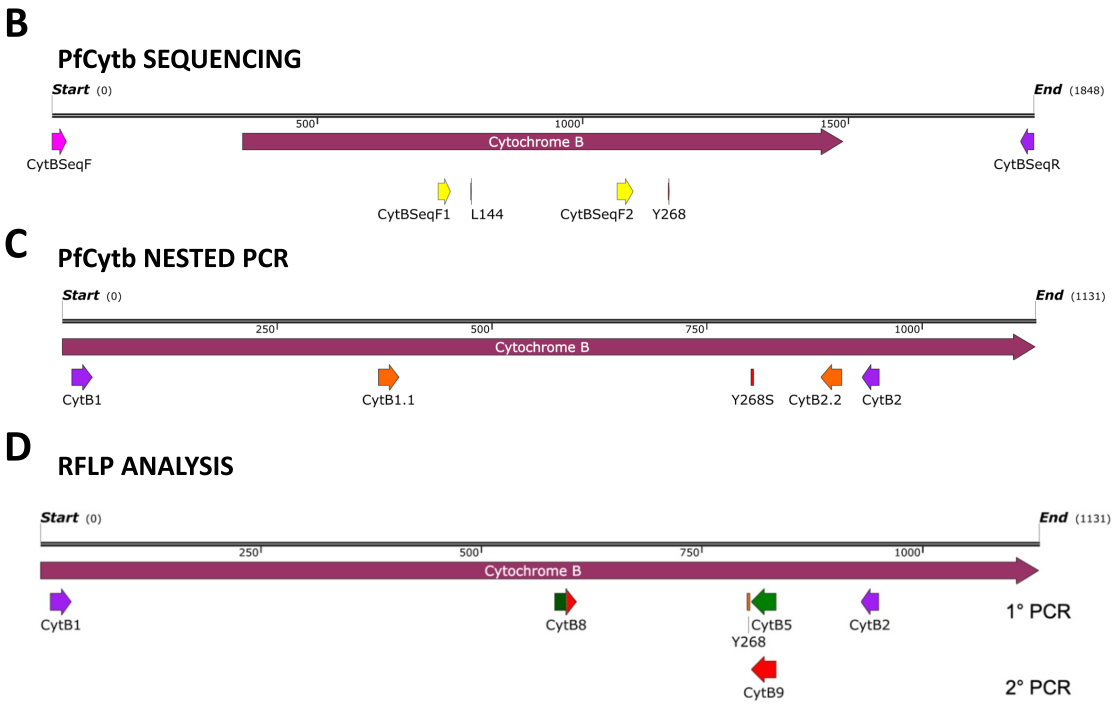

Supplementary Figure S2. PCR primers used in these studies. A Primer sequences and descriptions. **B** Graphic of primers for *Pfcytb* sequencing. **C** Graphic of primers for *Pfcytb* nested PCR. **D** Graphic of primers for RFLP analysis. To identify low levels of Y268S-encoding DNA in mosquito tissues, an RFLP method[2] was modified as follows.

DNA was isolated from midguts and salivary glands (Monarch Genomic DNA purification kit, NEB), and about 10 ng DNA was analyzed. In the primary PCR, a 939 bp product containing the mutation site was amplified with Taq DNA polymerase (NEB). Then 250 bp nested products were amplified by Taq DNA polymerase from 1 μL primary reaction product plus primer pairs: either CytB8 and CytB5 for detection of WT Y268, or CtyB8 and CytB9 for detection of mutant Y268S. Reverse primer CytB5, in conjunction with WT sequence TAT in the template DNA, results in an *Nsi*I recognition site (ATGCAT) in the nested PCR product. Reverse primer CytB9, in conjunction with mutant sequence TCT in the template, generates a *Pst*I recognition sequence (CTGCAG). For RFLP analysis, 5 μL of amplified DNA was digested (37 °C, overnight) with 1U *Nsi*I or *Pst*I (NEB). Products were resolved in 3% agarose and visualized by GelStar (Lonza) fluorescence. Successful digestion by either *Nsi*I or *Pst*I yields 224 and 26 bp products. From mixed cultures, an allelic population as low as 2% (*Nsi*I) or 10% (*Pst*I) can be detected.

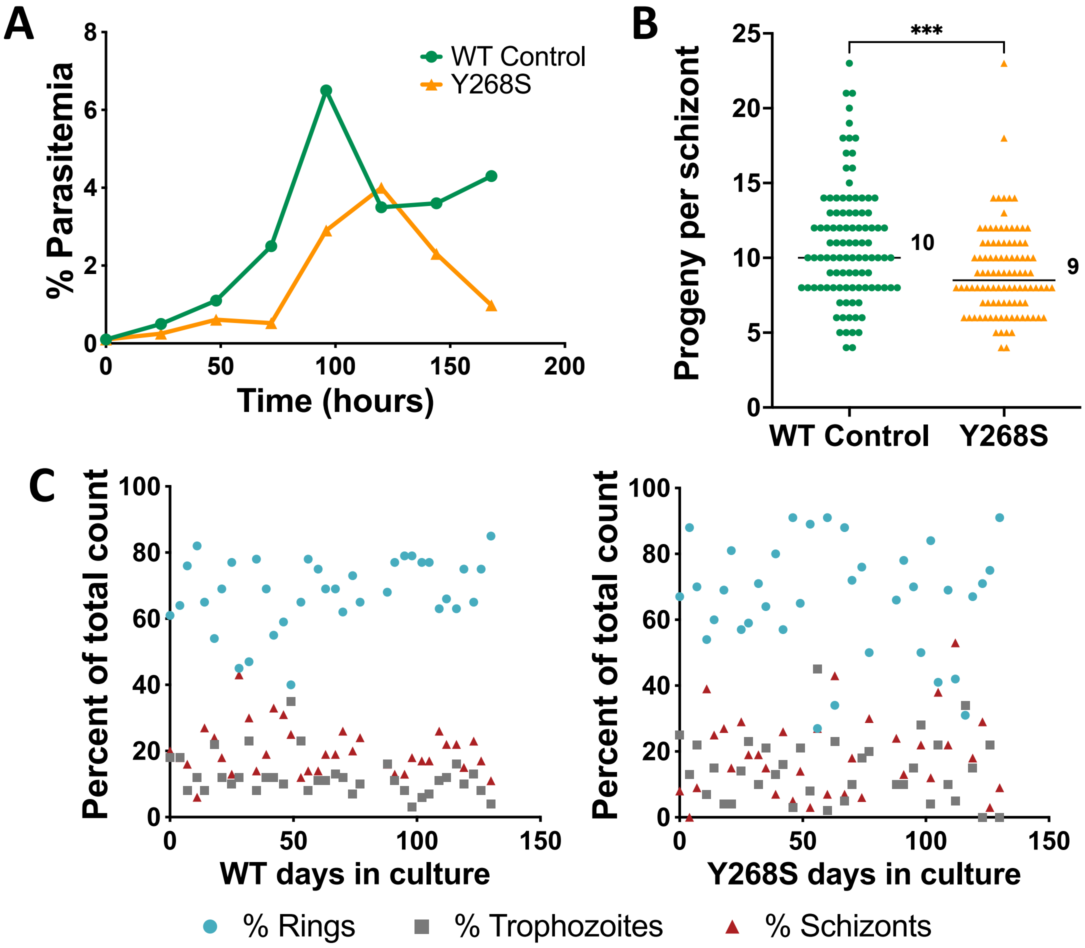

Supplementary Figure S3. Growth characteristics *in vitro* of WT and cytochrome *b* Y268S asexual erythrocytic parasites, obtained from one biological replicate. **A** Cell count was monitored at indicated intervals for a culture seeded at 0.1% parasitemia and not passaged. Relative to WT, mutant cells had a prolonged lag phase, delayed and lower peak parasitemia, and did not persist in stationary phase. Each point is the average of two replicate counts. **B** Progeny within late schizonts were counted in SYBR Gold-stained thin smear by fluorescence microscopy at 1000x magnification. Indicated are median values (ranges 4 to 23 for WT, and 4 to 23 for Y268S); $n = 100$, $***P = 0.0001$, two-sided Mann-Whitney test, single comparison. **C** Asynchronous parasites grown continuously over four months and without shaking were sampled for differential count at every 3-4 day passage. Depicted are percent of each stage in WT (*left panel*) and Y268S mutant (*right panel*) cultures. The tendency in WT cultures for trophozoites to be the least abundant form is lost in mutant populations. This alteration suggests that in mutants the maturation of metabolically active trophozoites is prolonged, a fitness cost consistent with impaired pyrimidine synthesis as a consequence of cytochrome *b* mutation. Source data are provided as a Source Data file.

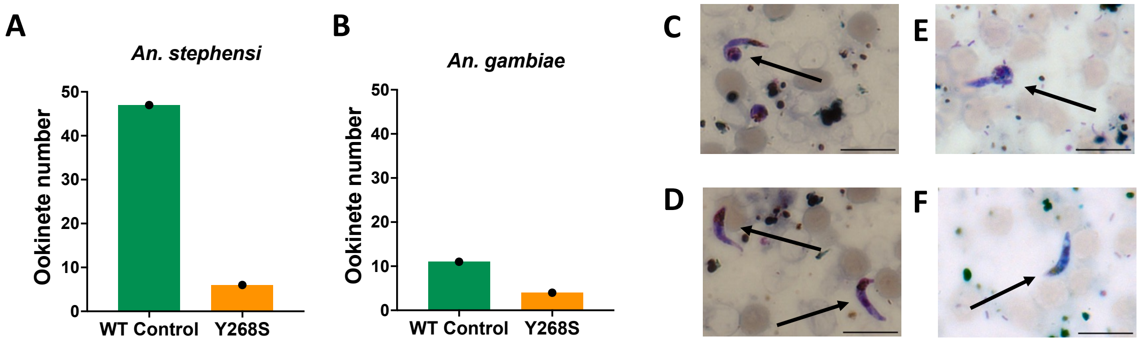

Supplementary Figure S4. Evaluation of ookinetes in anopheline mosquito midguts at 20-26 h after membrane feed. **A** Sum of mature and immature ookinetes in pooled samples obtained from ten *An. stephensi* midguts, in 60 independent 1000X microscopy fields. Data are from one biological experiment. **B** Sum of mature and immature ookinetes seen in ten *An. gambiae* midguts, in 60 independent 1000X microscopy fields. Data are from one biological experiment. **C** Immature WT control ookinete in *An. stephensi*. **D** Mature WT control ookinetes in *An. stephensi*. **E** Immature WT control ookinete in *An. gambiae*. **F** Mature WT control ookinetes in *An. gambiae*. *Bars*, 10 µm. *Arrows*, representative ookinetes. Source data are provided as a Source Data file.

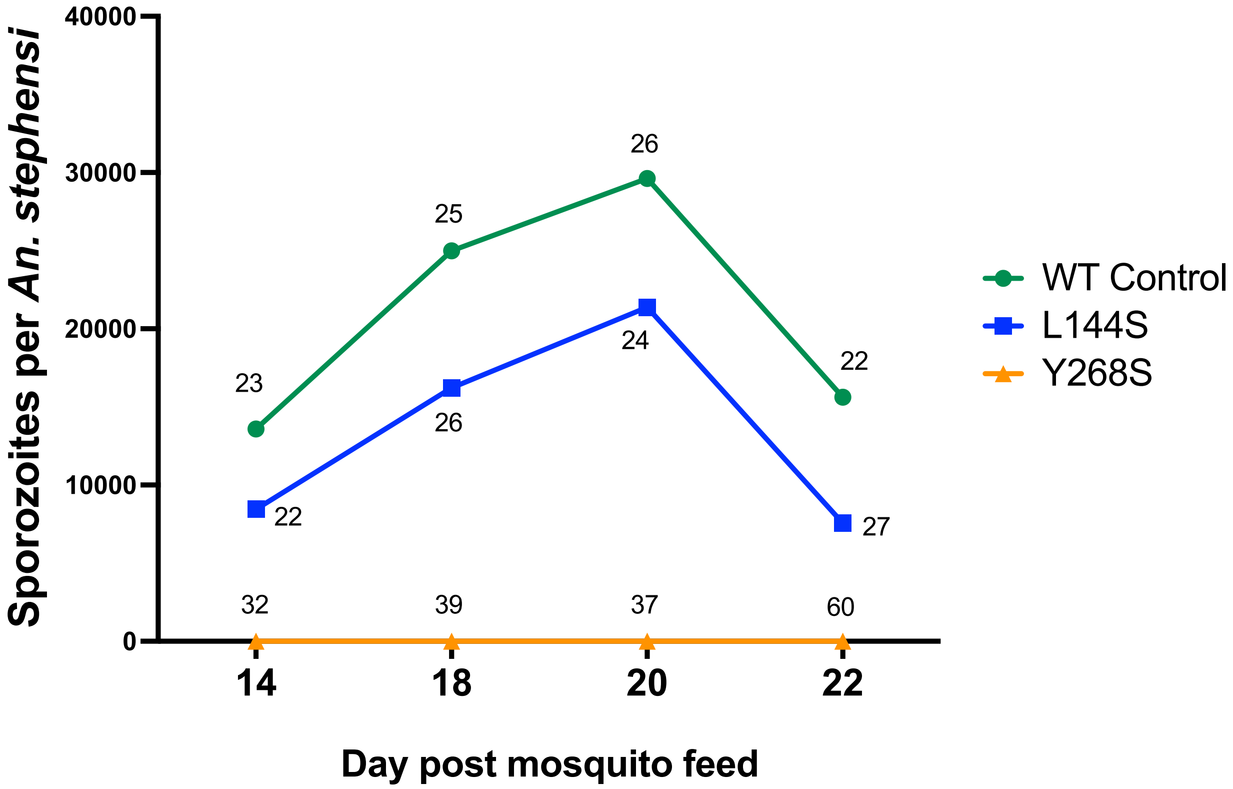

Supplementary Figure S5. ***An. stephensi*** **salivary gland sporozoite loads over time for wild type or cytochrome** ***b*** **mutant** ***P. falciparum*****.** At indicated intervals after bloodmeal, salivary glands were harvested and sporozoite load was assessed. Data are from one biological replicate. Number of mosquitoes dissected per timepoint for each experimental group is annotated, totaling 96 for WT, 99 for L144S, and 168 for Y268S. Source data are provided as a Source Data file.

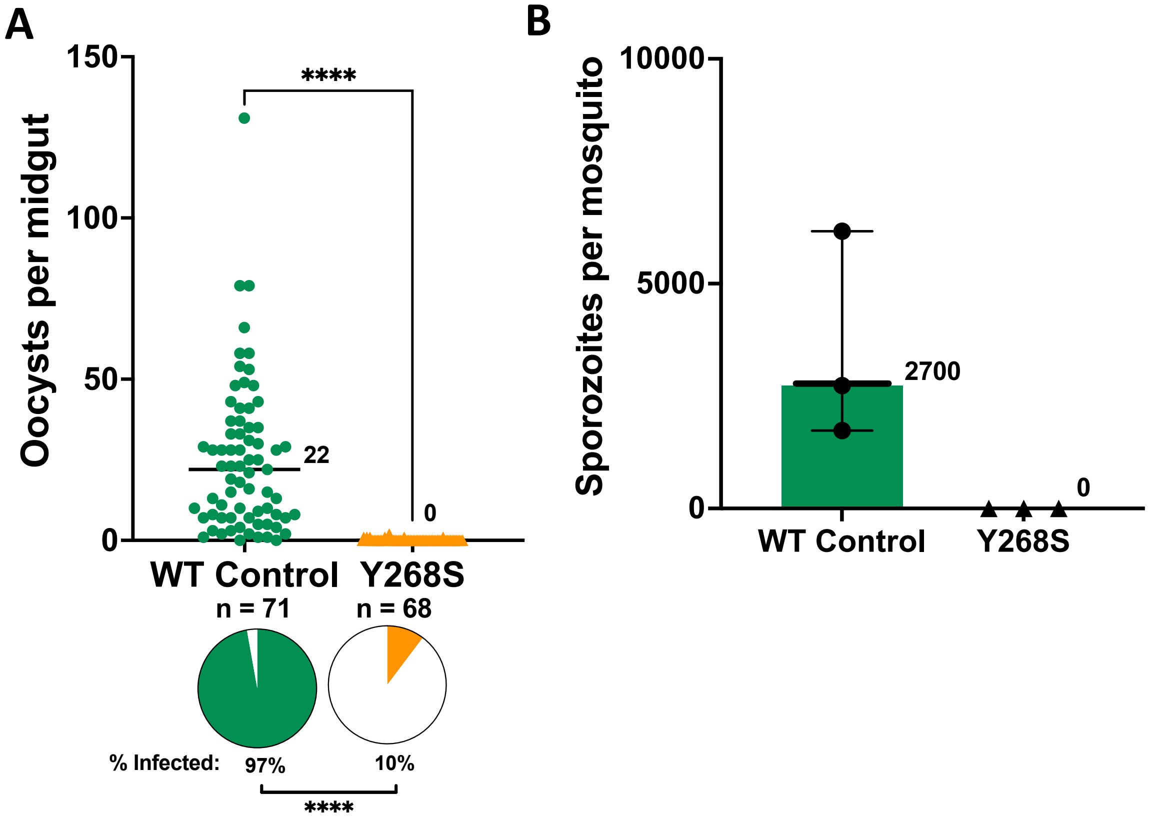

Supplementary Figure S6. *P. falciparum* counts in tissues of *An. gambiae*, from three biological replicate experiments. **A** Midguts were examined at 7-10 d after membrane feed. Median number of oocysts per midgut is indicated; ranges 0 to 131 for WT, or 0 to 2 for Y268S; *n*, total number of mosquitoes dissected; ****P <0.0001, two-sided Mann-Whitney test, single comparison; *pie charts,* percent of mosquitoes infected, ****P <0.0001, two-sided Fisher's exact test. **B** Salivary glands were dissected 14-19 d after membrane feed, from a total of 90 mosquitoes infected with WT or 100 infected with Y268S mutant parasites. *Symbols*, for each experiment, average number of sporozoites per mosquito. *Bars*, median values (indicated) and ranges (1700 to 6200 for WT; no sporozoites were seen in any Y268S sample); not powered for statistical analysis. Source data are provided as a Source Data file.

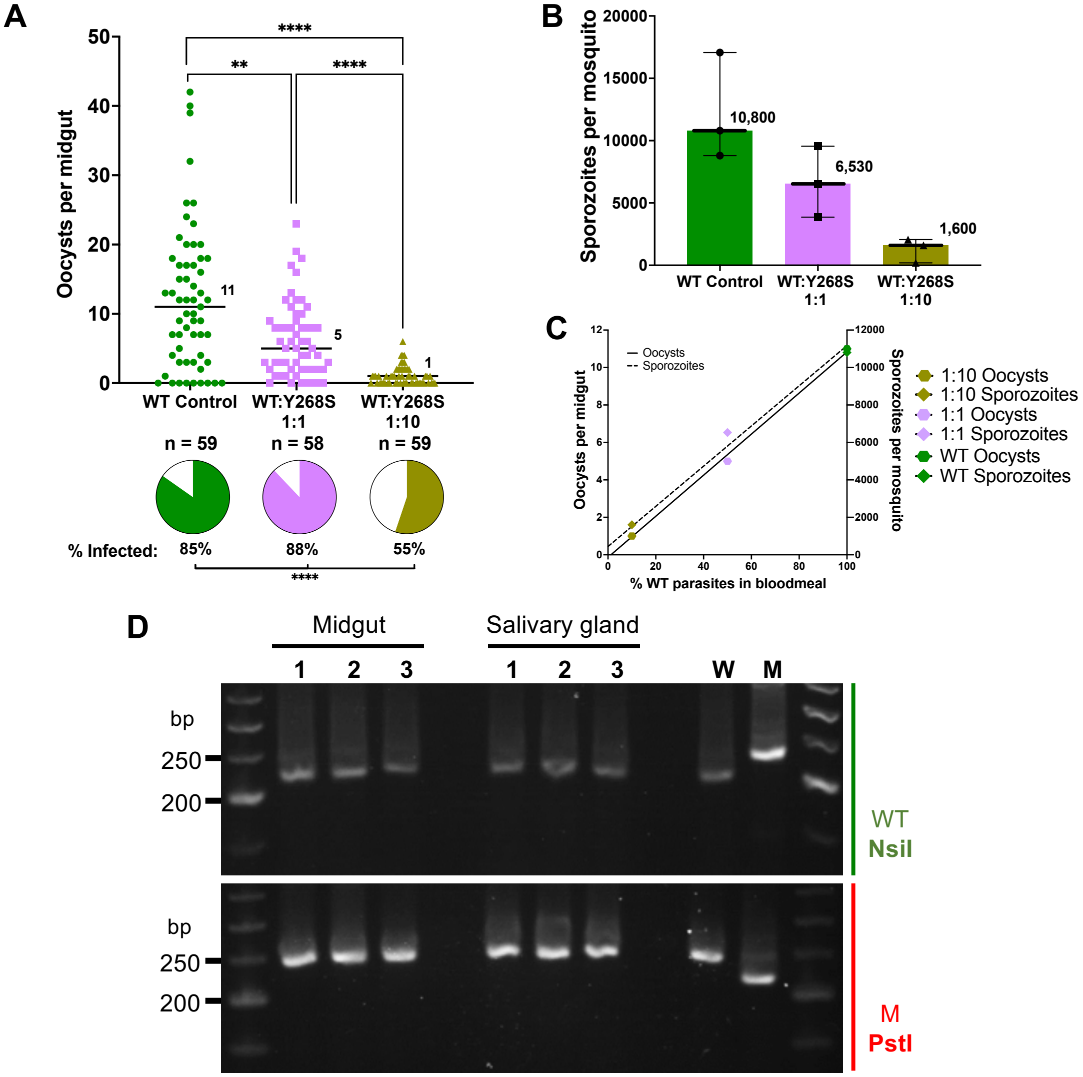

Supplementary Figure S7. Mixed infection with WT and Y268S mutant *P. falciparum* in *An. stephensi* mosquitoes, from three biological replicate experiments. Parasites were fed to mosquitoes in three cohorts: WT control (0.5% gametocytemia), a 1:1 mixture of WT:Y268S (0.25% WT:0.25% Y268S gametocytemia), or a 1:10 mixture of WT:Y268S (0.05% WT:0.45% Y268S gametocytemia). Results are from three independent biological experiments. **A** Oocysts were counted at 7-9 d after membrane feed. *Bars* are median (values indicated); WT range 0 to 42, 1:1 range 0 to 23, 1:10 range 0 to 6; *n*, number of mosquitoes dissected. **$P = 0.004$, ****$P < 0.0001$ by Kruskal-Wallis. *Pie charts* are percent of mosquitoes infected; ****$P < 0.0001$ by Chi-square. **B** Salivary glands were removed for sporozoite counts 14-16 d after infection. Total number of mosquitoes were 90 for WT, 91 for 1:1, and 90 for 1:10. *Symbols*, average sporozoites/mosquito for each experiment; *bars*, median (values indicated) and ranges (8,800 to 17,000 for WT; 3,900 to 9,500 for 1:1; 200 to 2,100 for 1:10). Overall Chi-square 0.039; not powered for further analysis. **C** Parasite numbers as a function of percent WT gametocytes in the bloodmeal. For oocysts $y = 0.11x - 0.14$, R^2 0.998; for sporozoites $y = 107x + 440$, R^2 0.988. **D** RFLP analysis for Y268S mutant DNA in mosquito tissues, obtained in three independent

biological experiments, each assayed in two independent technical replicates. *Upper panel*, *Nsi*I cleaves only the WT *Pfcytb* sequence, whereas *Pst*I cleaves only the sequence encoding Y268S (*lower panel*). In both cases a 224 bp product is released, allowing detection of an allelic population as low as 2% (*Nsi*I) or 10% (*Pst*I) (Supplementary Fig. S2A,D). *1*, WT control; *2*, WT:Y268S at 1:1; *3*, WT:Y268S at 1:10; *W*, WT DNA as assay control; *M*, Y268S DNA as assay control. Source data are provided as a Source Data file.

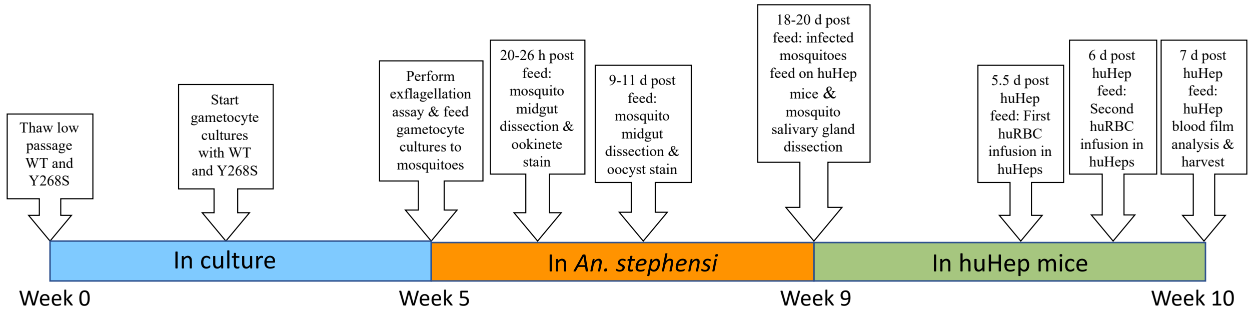

Supplementary Figure S8. Representative experimental timeline of *P. falciparum* transmission from *An. stephensi* to huHep mice.

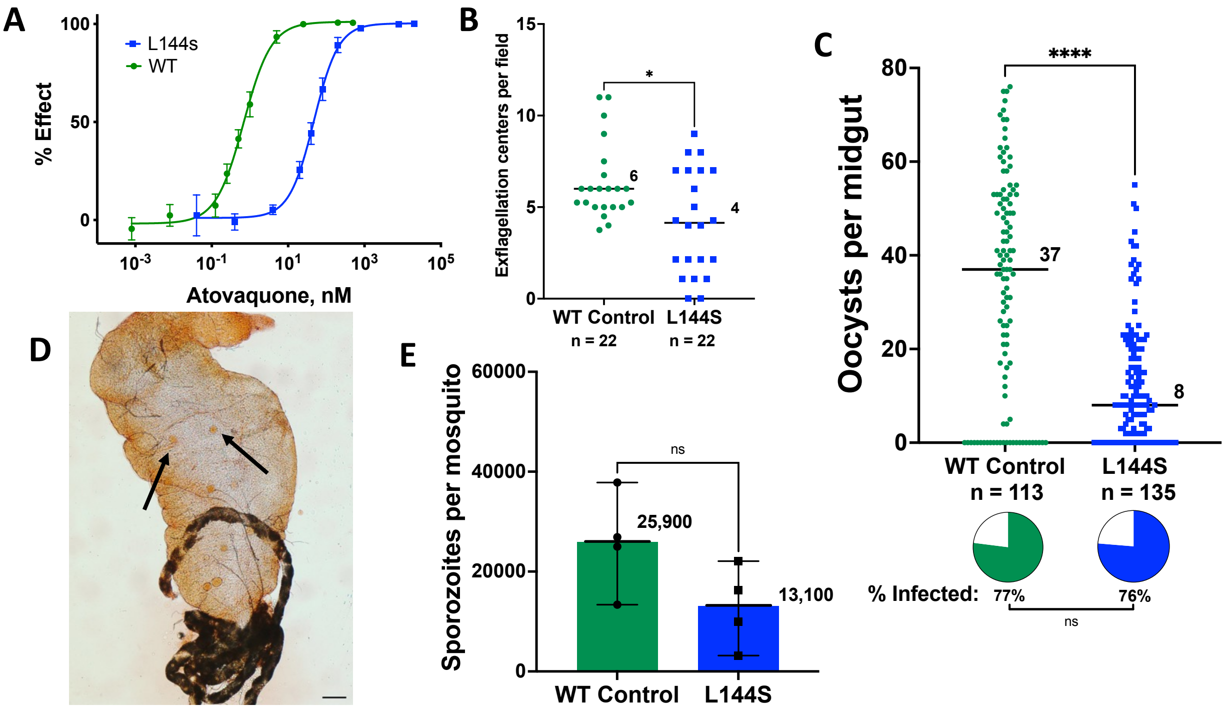

Supplementary Figure S9. Evaluation of paired wild type and L144S *P. falciparum in vitro* and in *An. stephensi* mosquitoes. **A** Atovaquone cytotoxicity against WT or L144S asexual erythrocytic parasites (EC_{50} 0.68 or 48 nM, respectively). Depicted are mean ± SD of quadruplicate determinations in each of three independent biological experiments (total $n = 12$; some SD are too small to extend outside the symbols); $R^2 \geq 0.997$. Susceptibilities of WT and L144S mutant cells to artemisinin (9.8 versus 8.5 nM) or chloroquine (8.2 versus 8.8 nM) were not significantly different. **B** Male gametocyte exflagellation adjusted to 1.5% gametocytemia. Median numbers in two independent biological replicates are indicated (ranges 4 to 11 for WT, and 0 to 9 for mutant); *n*, total number of fields examined; **P* = 0.0253, two-sided Mann-Whitney test, single comparison. **C** Oocysts in four independent biological replicates at 9-10 d after membrane feed. Median number of oocysts per midgut is indicated (ranges 0 to 76 for WT, and 0 to 55 for L144S); *n*, total number of mosquitoes dissected; *****P* <0.0001, two-sided Mann-Whitney test, single comparison; *pie charts* percent of midguts infected, two-sided Fisher's exact test. **D** Photomicrograph of representative midgut sample from *An. stephensi* infected with L144S *P. falciparum* gametocytes. *Bar*, 100 µm; a*rrow*, L144S oocyst. **E** *P. falciparum* sporozoite counts in four independent biological replicates at 17-20 d after membrane feed, entailing a total of 109 WT-fed and 118 mutant-fed mosquitoes. *Symbols*, for each experiment, average number of sporozoites per mosquito; *bars*, median number of sporozoites (indicated) and ranges 13,000 to 38,000 for WT; 3 to 22,000 for mutant); *P* = 0.1143, two-sided Mann-Whitney test, single comparison. Source data are provided as a Source Data file.

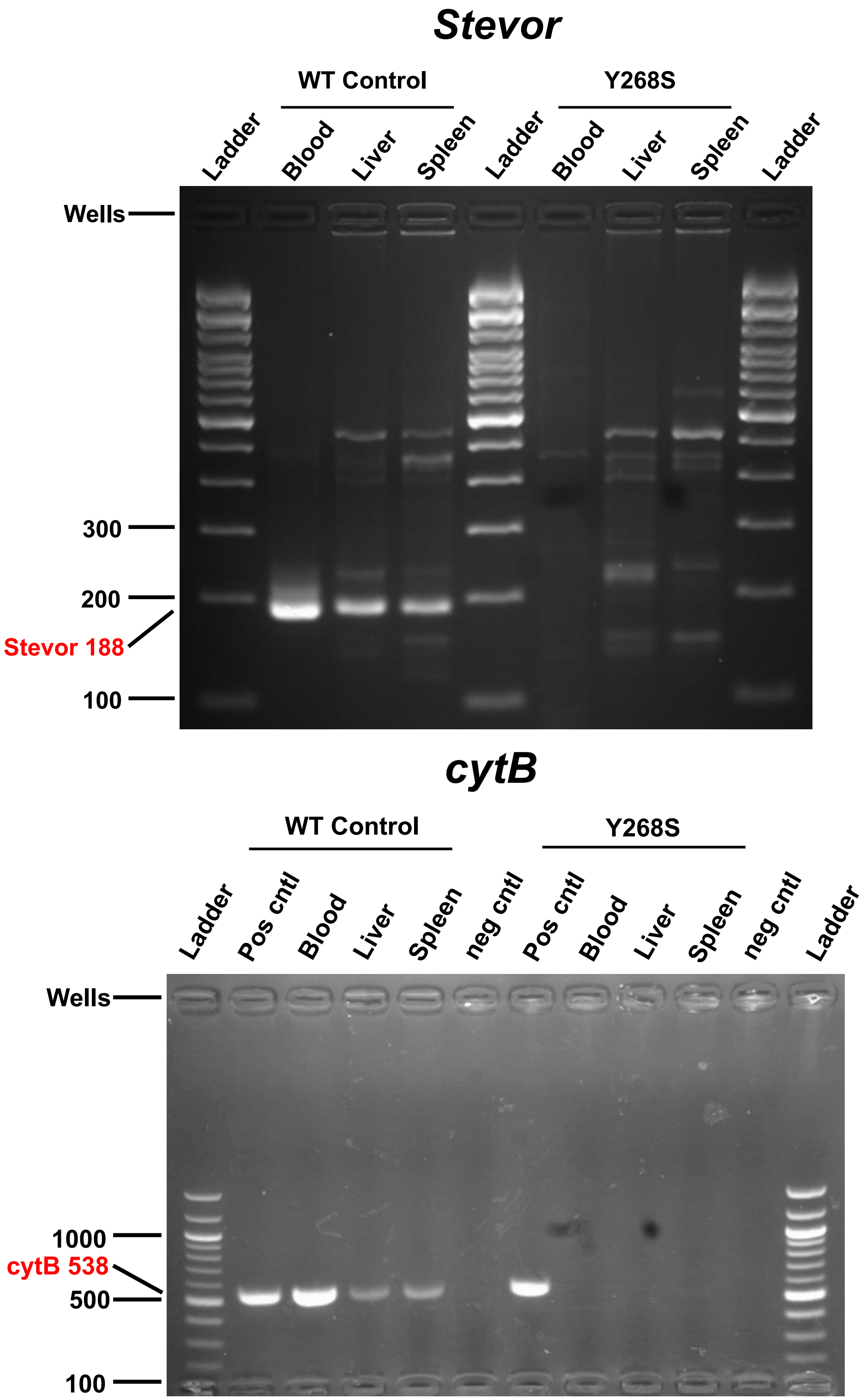

Supplementary Figure S10. Ten-fold longer exposures of manuscript Fig. 3 Panels B and C. Both panels are cropped to include gel images spanning from wells to 100 bp marker. Nonspecific PCR products are evident in *Stevor* assays, but only WT control

samples have a band of the expected size for the *Stevor* fragment. Full experimental details are provided in the legends for Figs. 3b and c.

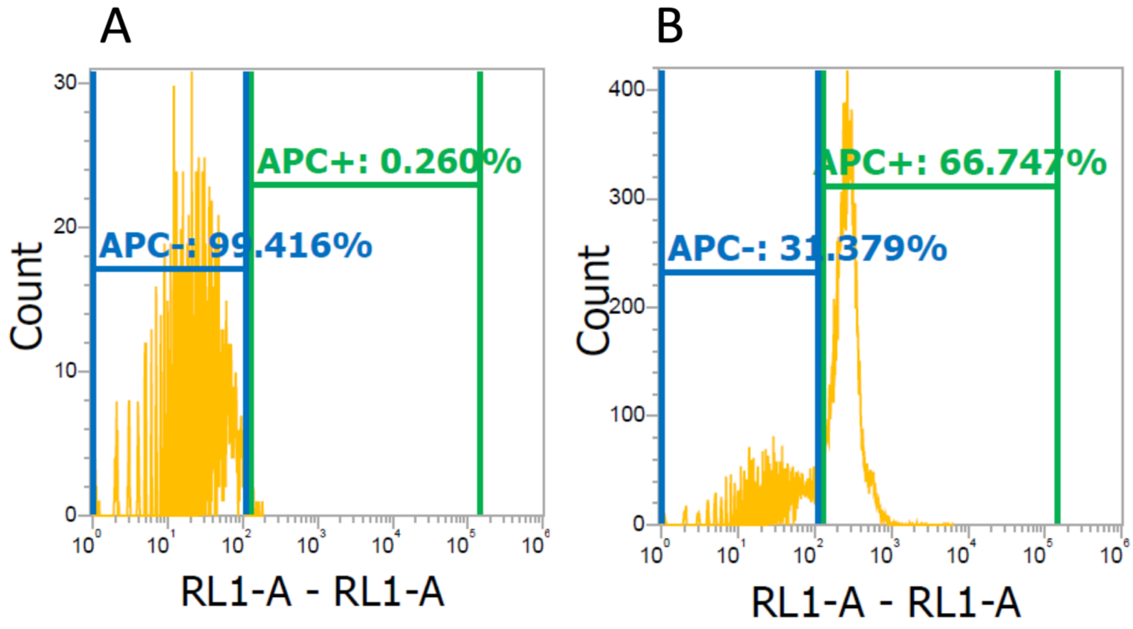

Supplementry Figure S11. Determination of human red cell engraftment in huHep mice. Blood collected from mice was treated with no antibody or with APC-conjugated rat anti-mouse glycophorin monoclonal antibody prior to separation by flow cytometry. **A** Unstained control sample showing both human and mouse erythrocytes within the APC- gate. **B** Similar analysis of blood incubated with antibody prior to cytometry distinguishes between human (APC-) and mouse (APC+) erythrocytes.

References

1. Shapiro, T. A., Ranasinha, C. D., Kumar, N., P. Barditch-Crovo, P. Prophylactic activity of atovaquone against *Plasmodium falciparum* in humans. *Am. J. Trop. Med. Hyg*. **60**, 831-836 (1999).

2. Schwöbel, B., Alifrangis, M., Salanti A., Jelinek, T. Different mutation patterns of atovaquone resistance to *Plasmodium falciparum in vitro* and *in vivo*: Rapid detection of codon 268 polymorphisms in the cytochrome b as potential *in vivo* resistance marker. *Malar J.* **2**, 5 (2003).
