# Supplementary material for: Clinically relevant atovaquone-resistant human malaria parasites fail to transmit by mosquito
Source: Nat Commun. 2023 Oct 12;14:6415. doi: 10.1038/s41467-023-42030-x (PMC10570281; doi:10.1038/s41467-023-42030-x)
Supplement: Supplementary file 3 — Description of Additional Supplementary Files [file 41467_2023_42030_MOESM3_ESM.pdf]

Description of Additional Supplementary Files

File Name: Supplementary Data 1
Description: Variants in whole genome sequence of *P. falciparum* Y268S cell line when compared to reference *P. falciparum* NF54 genome in PlasmoDB. VarQual Score = -10 Log_{10} P, where P is the probability of an incorrect base call.
