# Supplementary material for: Clinically relevant atovaquone-resistant human malaria parasites fail to transmit by mosquito
Source: Nat Commun. 2023 Oct 12;14:6415. doi: 10.1038/s41467-023-42030-x (PMC10570281; doi:10.1038/s41467-023-42030-x)
Supplement: Supplementary file 5 — Reporting Summary [file 41467_2023_42030_MOESM5_ESM.pdf]

nature portfolio

Corresponding author(s): Theresa A. Shapiro

Last updated by author(s): Sep 12, 2023

Reporting Summary

Nature Portfolio wishes to improve the reproducibility of the work that we publish. This form provides structure for consistency and transparency in reporting. For further information on Nature Portfolio policies, see our Editorial Policies and the Editorial Policy Checklist.

Statistics

For all statistical analyses, confirm that the following items are present in the figure legend, table legend, main text, or Methods section.

n/a	Confirmed	
☐	☒	The exact sample size (*n*) for each experimental group/condition, given as a discrete number and unit of measurement
☐	☒	A statement on whether measurements were taken from distinct samples or whether the same sample was measured repeatedly
☐	☒	The statistical test(s) used AND whether they are one- or two-sided<br>*Only common tests should be described solely by name; describe more complex techniques in the Methods section.*
☒	☐	A description of all covariates tested
☒	☐	A description of any assumptions or corrections, such as tests of normality and adjustment for multiple comparisons
☐	☒	A full description of the statistical parameters including central tendency (e.g. means) or other basic estimates (e.g. regression coefficient) AND variation (e.g. standard deviation) or associated estimates of uncertainty (e.g. confidence intervals)
☐	☒	For null hypothesis testing, the test statistic (e.g. *F*, *t*, *r*) with confidence intervals, effect sizes, degrees of freedom and *P* value noted<br>*Give P values as exact values whenever suitable.*
☒	☐	For Bayesian analysis, information on the choice of priors and Markov chain Monte Carlo settings
☒	☐	For hierarchical and complex designs, identification of the appropriate level for tests and full reporting of outcomes
☒	☐	Estimates of effect sizes (e.g. Cohen's *d*, Pearson's *r*), indicating how they were calculated

Our web collection on statistics for biologists contains articles on many of the points above.

Software and code

Policy information about availability of computer code

Data collection	Microsoft Excel 16.73; Attune Nxt Software
Data analysis	GraphPad Prism 6 or 9; NCI BLAST, SnapGene 7.0, Partek Flow 10.0.0410, Bowtie2 2.2.5, FreeBayes, Microsoft Excel 16.73, ImageJ 1.53

For manuscripts utilizing custom algorithms or software that are central to the research but not yet described in published literature, software must be made available to editors and reviewers. We strongly encourage code deposition in a community repository (e.g. GitHub). See the Nature Portfolio guidelines for submitting code & software for further information.

Data

Policy information about availability of data

All manuscripts must include a data availability statement. This statement should provide the following information, where applicable:

- Accession codes, unique identifiers, or web links for publicly available datasets
- A description of any restrictions on data availability
- For clinical datasets or third party data, please ensure that the statement adheres to our policy

All data supporting the findings of this study are provided in the paper and its supplementary information. The minimum dataset necessary to interpret, verify and extend the research in this article are provided in the Supplementary Information/Source Data file. Whole genome sequence files for WT parent and Y268S mutant have been deposited in the NCBI Sequence Read Archive (BioProject ID: PRJNA913198; https://www.ncbi.nlm.nih.gov/sra).

Research involving human participants, their data, or biological material

Policy information about studies with human participants or human data. See also policy information about sex, gender (identity/presentation), and sexual orientation and race, ethnicity and racism.

Reporting on sex and gender	*Use the terms sex (biological attribute) and gender (shaped by social and cultural circumstances) carefully in order to avoid confusing both terms. Indicate if findings apply to only one sex or gender; describe whether sex and gender were considered in study design; whether sex and/or gender was determined based on self-reporting or assigned and methods used.*<br>*Provide in the source data disaggregated sex and gender data, where this information has been collected, and if consent has been obtained for sharing of individual-level data; provide overall numbers in this Reporting Summary. Please state if this information has not been collected.*<br>*Report sex- and gender-based analyses where performed, justify reasons for lack of sex- and gender-based analysis.*
Reporting on race, ethnicity, or other socially relevant groupings	*Please specify the socially constructed or socially relevant categorization variable(s) used in your manuscript and explain why they were used. Please note that such variables should not be used as proxies for other socially constructed/relevant variables (for example, race or ethnicity should not be used as a proxy for socioeconomic status).*<br>*Provide clear definitions of the relevant terms used, how they were provided (by the participants/respondents, the researchers, or third parties), and the method(s) used to classify people into the different categories (e.g. self-report, census or administrative data, social media data, etc.)*<br>*Please provide details about how you controlled for confounding variables in your analyses.*
Population characteristics	*Describe the covariate-relevant population characteristics of the human research participants (e.g. age, genotypic information, past and current diagnosis and treatment categories). If you filled out the behavioural & social sciences study design questions and have nothing to add here, write "See above."*
Recruitment	*Describe how participants were recruited. Outline any potential self-selection bias or other biases that may be present and how these are likely to impact results.*
Ethics oversight	*Identify the organization(s) that approved the study protocol.*

Note that full information on the approval of the study protocol must also be provided in the manuscript.

Field-specific reporting

Please select the one below that is the best fit for your research. If you are not sure, read the appropriate sections before making your selection.

[x] Life sciences [ ] Behavioural & social sciences [ ] Ecological, evolutionary & environmental sciences

For a reference copy of the document with all sections, see nature.com/documents/nr-reporting-summary-flat.pdf

Life sciences study design

All studies must disclose on these points even when the disclosure is negative.

Sample size	Sample sizes were determined prospectively based on results from similar experiments in our previous projects, or in literature reports (PMID 22911675, 21262960, 19064257, 18346224, 15630135) or in a methods chapter on these procedures (PMID 22990793.
Data exclusions	Data were excluded from two experiments (infection of mosquitoes by wt or mutant parasites) because wt control results were more than two standard deviations below historical means, and the mosquito colony was found to be infected with a fungus.
Replication	For all experiments, the number of biological and technical replicates is now provided in the manuscript and its Supplementary Information. All key experiments were conducted in 2-9 independent biological replicates. Some experiments, all in Supplementary Information, were done just once. All attempts at replication were successful except as indicated above in Data exclusions, when mosquitoes were not healthy.
Randomization	All samples were, of necessity, allocated to just one of two experimental groups: wild type or mutant parasites. These experiments were descriptive and did not include any interventions. Every independent replicate included both experimental groups, so as to control for the many variables inherent in these biological studies.
Blinding	Data were acquired in a blinded fashion for Figs. 1c and 3a. Vivid differences between wild type and mutant parasites precluded blinding for many of the experiments. Five different laboratories contributed to this project, some are well-versed in blinding and have procedures in place to prevent sample mixup. Others, however, are still in the process of establishing these methods.

Reporting for specific materials, systems and methods

We require information from authors about some types of materials, experimental systems and methods used in many studies. Here, indicate whether each material, system or method listed is relevant to your study. If you are not sure if a list item applies to your research, read the appropriate section before selecting a response.

Materials & experimental systems

n/a	Involved in the study
[ ]	[x] Antibodies
[ ]	[x] Eukaryotic cell lines
[x]	[ ] Palaeontology and archaeology
[ ]	[x] Animals and other organisms
[x]	[ ] Clinical data
[x]	[ ] Dual use research of concern
[x]	[ ] Plants

Methods

n/a	Involved in the study
[x]	[ ] ChIP-seq
[ ]	[x] Flow cytometry
[x]	[ ] MRI-based neuroimaging

Antibodies

Antibodies used	APC-conjugated TER-119 rat anti-mouse monoclonal antibody (BD Biosciences catalog no 557909, lot 9213638)
Validation	Method for using these antibodies to quantify mouse RBCs in human RBC-engrafted mice is described in Jimenez Diaz MB, Mulet T, Viera S, Gomez V, Garuti H, Ibanez J, Alvarez-Doval A, Shultz LD, Martinez A, Gargallo-Viola D, AnguloBarturen I (2009) Improved murine model of malaria using Plasmodium falciparum competent strains and non-myelodepleted NODscid IL2Rgammanull mice engrafted with human erythrocytes. Antimicrob Agents Chemother 53:4533-6

Eukaryotic cell lines

Policy information about cell lines and Sex and Gender in Research

Cell line source(s)	Human malaria parasite isolate used in these studies was obtained through BEi Resources, NIAID, NIH: Plasmodium falciparum, Strain NF54 (Patient Line E) MRA-1000, contributed by Megan G. Dowler.<br>The E line of NF54 stock parasites was amplified in a volunteer patient "E", who participated in a clinical trial in 1995 at Walter Reed Army Institute of Research. The parent NF54 strain of Plasmodium falciparum was isolated from a patient living near an airport in the Netherlands, who had never left the country.
Authentication	Strain NF54 "E" line has been minimally passaged in vitro and produces competent gametocytes for mosquito (Anopheles stephensi) infection up to passage 40. The complete genome of the parent strain P. falciparum NF54, has been sequenced (GenBank: AMYQ00000000).
Mycoplasma contamination	No mycoplasma was detected in these cell lines.
Commonly misidentified lines<br>(See ICLAC register)	Not applicable

Animals and other research organisms

Policy information about studies involving animals; ARRIVE guidelines recommended for reporting animal research, and Sex and Gender in Research

Laboratory animals	FRG LP (Faj-/-, Rag2-/-, IL2rg-/-) on NOD background; 27-28 wks old
Wild animals	Study did not include wild animals.
Reporting on sex	All experiments were conducted in female mice. Any male-female differences in mouse models of malaria are subtle, and would require a large number of animals to assess. The huHep mice we studied are rare, fragile to maintain, and costly (thousands of dollars per mouse), so an evaluation of sex as a biological variable was not felt to be within the scope of this study.
Field-collected samples	Study did not involve samples collected from the field.
Ethics oversight	The Johns Hopkins University Animal Care and Use Committee

Note that full information on the approval of the study protocol must also be provided in the manuscript.

Flow Cytometry

Plots

Confirm that:

- [x] The axis labels state the marker and fluorochrome used (e.g. CD4-FITC).
- [x] The axis scales are clearly visible. Include numbers along axes only for bottom left plot of group (a 'group' is an analysis of identical markers).
- [ ] All plots are contour plots with outliers or pseudocolor plots.
- [x] A numerical value for number of cells or percentage (with statistics) is provided.

Methodology

Sample preparation	Blood samples (2 µL) were taken from the lateral tail vein, transferred into 100 µL PBS containing 10 µg/mL antibody, and incubated for 20 min. Engraftment with hRBC was assessed as the percentate of APC- erythrocytes
Instrument	Thermo Scientific Attune Nxt Flow Cytometer
Software	Attune Nxt
Cell population abundance	Percent human RBC in mouse blood was obtained directly from APC staining signals.
Gating strategy	Mouse RBCs were stained with APC-conjugated antibodies so our gating strategy was simply APC+ (mouse) and APC- (human) RBCs.

- [x] Tick this box to confirm that a figure exemplifying the gating strategy is provided in the Supplementary Information.
